# Feasibility Evaluation of Radioimmunoguided Surgery of Breast Cancer

**DOI:** 10.1155/2012/545034

**Published:** 2012-02-20

**Authors:** Ananth Ravi, Raymond M. Reilly, Claire M. B. Holloway, Curtis B. Caldwell

**Affiliations:** ^1^Department of Medical Physics, Sunnybrook Odette Cancer Centre, 2075 Bayview Avenue, Toronto, ON, Canada M4N 3M5; ^2^Leslie Dan Faculty of Pharmacy, University of Toronto, Toronto, ON, Canada M5S 3M2; ^3^Department of Medical Imaging, University of Toronto, Toronto, ON, Canada M5S 3E2; ^4^Department of Surgery, Sunnybrook Health Sciences Centre, Toronto, ON, Canada M4N 3M5

## Abstract

Breast-conserving surgery involves completely excising the tumour while limiting the amount of normal tissue removed, which is technically challenging to achieve, especially given the limited intraoperative guidance available to the surgeon. This study evaluates the feasibility of radioimmunoguided surgery (RIGS) to guide the detection and delineation of tumours intraoperatively. The 3D point-response function of a commercial gamma-ray-detecting probe (GDP) was determined as a function of radionuclide (^131^I, ^111^In,^ 99m^Tc), energy-window threshold, and collimator length (0.0–3.0-cm). This function was used to calculate the minimum detectable tumour volumes (MDTVs) and the minimum tumour-to-background activity concentration ratio (T:B) for effective delineation of a breast tumour model. The GDP had larger MDTVs and a higher minimum required T:B for tumour delineation with ^131^I than with ^111^In or ^99m^Tc. It was shown that for ^111^In there was a benefit to using a collimator length of 0.5-cm. For the model used, the minimum required T:B required for effective tumour delineation was 5.2 ± 0.4. RIGS has the potential to significantly improve the accuracy of breast-conserving surgery; however, before these benefits can be realized, novel radiopharmaceuticals need to be developed that have a higher specificity for cancerous tissue *in vivo *than what is currently available.

## 1. Introduction

In North America, over 60% of breast cancer patients receive breast-conserving surgery [1]. The primary goal of this operative procedure is the complete excision of the cancerous lesion, with a margin of grossly normal tissue. The purpose of this margin is to reduce the probability that microscopic disease remains. A secondary, conflicting goal is to limit the volume of normal tissue that is excised, thereby reducing patient morbidity and improving cosmesis. Achieving these goals is technically challenging, and incomplete excision occurs in 15–40% of breast-conserving operations, with pathologic evaluation revealing cancerous cells at the cut edge of the excised volume [2, 3]. Studies by Park et al. [4] and Peterson et al. [5] have independently shown that tumours with diameters greater than 2 cm have a higher likelihood of involved margins than smaller tumours. This may be attributed to the presence of nonpalpable disease at the boundaries of these tumours. This largely intra-ductal disease is difficult to detect using currently available guidance techniques that rely on anatomical differences between normal tissue and tumour [2, 6]. 

There is currently only limited intraoperative guidance available for breast procedures. For palpable tumours, the palpable edge is used to guide the complete excision of the tumour. For nonpalpable tumours, wire localization is the standard procedure. During wire placement, radiographic images are obtained to guide the surgeon; however, the wire may shift position within the breast, providing misleading information about the tumour location at the time of breast-conserving surgery. Additionally, the wire only marks the approximate location of the centre of the gross tumour within the breast and provides no information as to its extent. Competing techniques to wire localization are being developed; these techniques include radioguided occult lesion localization (ROLL) and radioactive seed localization (RSL). Radiographic or sonographic guidance is used to inject a ^99m^Tc labelled nanocolloidal tracer peritumorally in the case of ROLL or implant an ^125^I brachytherapy seed at the center of the nonpalpable tumour in the case of RSL. The surgeon then uses a hand-held gamma-ray-detecting probe (GDP), to guide the excision of the tumour [7–11]. Ultrasonography has been explored as an alternative approach; however, patients with tumours having extensive intraductal disease or a predominantly infiltrative growth pattern are not candidates for ultrasound-guided excision [12–14].

Radioimmunoguided surgery (RIGS) differentiates normal and cancerous tissues by exploiting the functional status of cancerous cells. One such functional property is the degree of overexpression of tumour-associated antigens (TAAs). Thus, a targeting vehicle specific to TAAs, such as a monoclonal antibody or an antibody fragment, can be conjugated to a radioactive label to create a radiopharmaceutical that molecularly targets cancerous tissues. For RIGS of breast cancer, TAAs that have been targeted by radiopharmaceuticals include tumour-associated glycoprotein-72 (TAG-72) [15], human epidermal growth factor-2 (HER-2) [16, 17], and carcinoembryonic antigen (CEA) [18–23].  Table 1 lists a sample of the radiopharmaceuticals that have been used to target TAAs in breast cancer. 

The clinical utilization of radioimmunoguidance involves the administration of a radiolabelled monoclonal antibody or antibody fragment two hours to several days prior to the breast-conserving surgery. This allows sufficient time for the radiopharmaceutical to distribute throughout the patient and attach preferentially to the TAAs overexpressed on cancer cells. At the time of the surgery, prior to making an incision and with the patient in the appropriate position, the surgical oncologist takes a measurement of normal background activity using GDP. The GDP is then used to detect the tumour, which is where the measured count rates are significantly greater than normal background count rates. By taking measurements along the skin surface, moving outward from the centre of the tumour, the surgeon can delineate tumour extent as the point where measured count rates are no longer significantly higher than background.

In this study, we address two fundamental issues that arise when considering the potential efficacy of RIGS. The first is that there is currently no reference minimum T:B to determine if a radiopharmaceutical would be a promising candidate. The T:B for a particular radiopharmaceutical is a function of several parameters such as its tumour-targeting ability, tumour perfusion, and normal tissue clearance [24, 25]. The second issue is that there is no consensus regarding which the optimum combination of device parameters for RIGS is. The two main components of a RIGS system that affect its performance and are within the control of the user are the radiopharmaceutical and the GDP. Important study parameters related to the radiopharmaceutical include the radionuclide used to label the targeting agent and the amount of activity administered to the patient. Important study parameters related to the GDP that affect its sensitivity and spatial resolution include the characteristics of the collimator, the degree of scattered radiation that is rejected by the device, and the measurement time. There is currently little relevant information in the literature on the technical requirements for optimizing RIGS for breast cancer.

This study reports on the results of a systematic phantom-based evaluation of the impact of various critical parameters (radionuclide, background activity concentration, measurement time, collimator length, energy-window threshold) on the minimum detectable tumour volume (MDTV) and comments on the feasibility of using RIGS for tumour detection and delineation during breast-conserving surgery.

## 2. Methods

### 2.1. Experimental Apparatus and Setup

**Figure 1:**
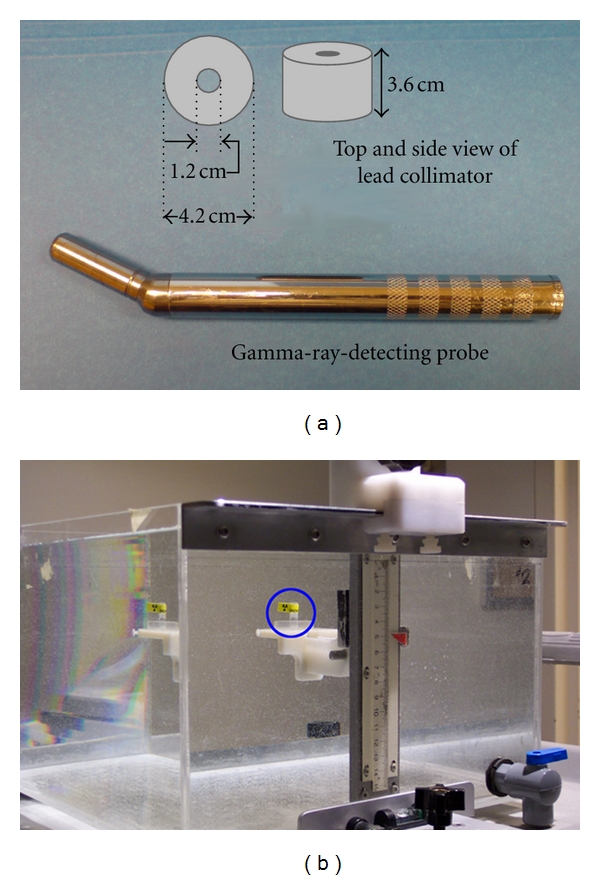
The experimental apparatus used to acquire the point-response function of the GDP for each radionuclide. (a) Photograph C-Trak NaI (Tl) probe (3 cm long, 1 cm diameter cylindrical crystal). Diagram of lead collimator 1.5 cm thick and 3.6 cm in length. (b) 8 mm^3^ source (circled in blue) in a water tank. The source can be displaced horizontally or in depth with 0.5 mm resolution.

The apparatus used for the experiments consisted of the C-Trak surgical guidance system (Care Wise Medical Products Corporation, California), with a 3.0 cm long and 1.0 cm diameter cylindrical NaI(Tl) crystal. A simple side shield was manufactured in-house as a 1.5 cm thick, 3.6 cm long hollow cylinder made of lead, so that design specifications could be controlled during the study. It was constructed to fit tightly around the sensitive end of the GDP and completely shielded the NaI(Tl) crystal except for the open face. The thickness of the side shield was chosen such that the penetration was limited to less than 5% at energies up to 364 keV. The side shield was designed to limit interference from photons emitted outside the nominal field of view of the detector. The face of the side shield was constructed so that it would be flushed with the sensitive end of the GDP; consequently, it did not provide any additional collimation. The GDP and the side shield were centred on a water tank phantom and were used to acquire the sensitivity map of a GDP that has robust side shielding, with 0.0 cm of additional collimation (Figure 1(a)).Within the water tank, a radioactive source could be translated horizontally and depth wise with respect to the probe position, with a translational precision of 0.5 mm. A water tank was used to simulate in the GDP sensitivity measurements the effect of photon scatter and attenuation expected in the breast. 

The radioactive source was composed of a polymethyl methacrylate capsule with a cavity in the shape of a 2 × 2 mm cylinder filled with radionuclide. The radioactive source was constructed to be of similar diameter to that of milk ducts found in the breast [26]. The wall thickness of the capsule was 1 mm. The three radionuclides used in this evaluation were ^99m^Tc, ^111^In, and ^131^I. These radionuclides are common radiolabels that have been proposed for use in RIGS [22, 27]. Each radionuclide emits gamma-rays of differing energies, allowing a range of photon energies to be evaluated, ^99m^Tc (140 keV, 89%), ^111^In (171 keV, 90%; 245 keV, 94%), and ^131^I (364 keV, 90%) [27].

The point-response function of the GDP was measured in the water tank phantom for each of the three radionuclides. The sources were moved to a total of 352 positions in a 2D plane, in three overlapping grids: a fine grid with a 2 mm spacing used for the region closest to the GDP where the sensitivity changes rapidly and coarser grid spacings of 5 mm and 10 mm successively further away from the GDP. The total region covered was 70 mm horizontally by 130 mm in depth. For each source position, an energy spectrum was acquired using a multichannel analyzer. A similar technique for characterizing probe response has been used by Kwo et al. [28] for only a single radionuclide ^57^Co.

To generate energy-dependent 2D point-response functions, an energy-window threshold was applied to the spectra at each source position. The energy-window thresholds to be evaluated were determined by dividing an energy range (from the Compton backscatter peak to three standard deviations past the photopeak) into ten evenly spaced intervals. The energy ranges that were investigated for each radionuclide were as follows: ^99m^Tc (90–200 keV), ^111^In (100–350 keV), and ^131^I (150–460 keV). Photons detected with energies below the Compton backscatter peak are more likely to be a result of multiple scatters in the patient rather than in the detecting crystal and may also arise from the characteristic X-rays emitted from the lead collimator, which contain no useful positional information, thus making the backscatter peak a suitable starting point for the energy range. The collimator response was then calculated and applied to the 2D point-response function, based on the formalism described by Barrett and Swindell [29]. The calculated collimator response assumed that the collimator was a perfect absorber and did not account for scattering that may occur within the collimator. A 3D point-response function for the GDP was generated by rotating the 2D map around the central axis of rotational symmetry for the GDP. The 3D point-response function could then be used to calculate the response of the GDP to any activity distribution for a particular radionuclide, energy-window threshold, and collimator length.

The 3D point-response function was validated experimentally by taking measurements of the radioactivity emitted by the capsule point source at 1 cm intervals along the horizontal axis, at a consistent depth of 1 cm, with a fixed collimator length of 0.5 cm and the energy-window threshold at Compton back scatter peak. This procedure was repeated for each of the radionuclides used in this study.

### 2.2. Minimum Detectable Tumour Volume (MDTV)

Current re-excision rates are 15–40%, which can be used as an estimate of the false-negative rates associated with lumpectomy procedures [2, 3]. If a RIGS technique could reduce this value, this would be of significant benefit to patients. This study asks whether or not it is possible for a RIGS technique to reduce the false-negative rate to 5%, which would be a significant improvement over what is currently achievable clinically. The number of counts that need to be detected in order to achieve a 5% false-negative and 5% false-positive rate given an estimated background activity can be calculated using the formalism described by Currie [30]. The number of counts above the mean background count that corresponds to a 5% false-positive and false-negative rates is referred to as the *a priori *detection limit L_D_:

(1) $$\begin{array}{cc} {\text{L}}_{\text{D}} & =2.71+4.65\sqrt{\mu_{\text{B}}} \end{array},$$

where *μ*_B_ is the mean number of counts measured for normal background tissue. In a simulation if a tumour model generated counts equal to or greater than the detection limit + mean background counts, the specificity and sensitivity would be 95% for detecting tumours of that size [30].

The bulk of the normal background activity was assumed to arise from the blood supply within the breast (given that the radiopharmaceutical was administered intravenously). This was not an ideal model as it did not incorporate nonspecific distribution of the radiopharmaceutical within the interstitium. However, nonspecific distribution is poorly characterized in the literature and, in the absence of relevant clinical data, the foregoing phantom geometry is likely a reasonable approximation of that encountered *in vivo*. A practical range of background activity concentration from 1 − 5^kBq^/ml was used in this study [15, 20, 22]. The background activity concentration of breast tissue was calculated by multiplying the background activity concentration by the volume of blood within breast tissue (0.0336 ± 0.006_mL  of  blood/mL  of  tissue_) [31].

**Figure 2:**
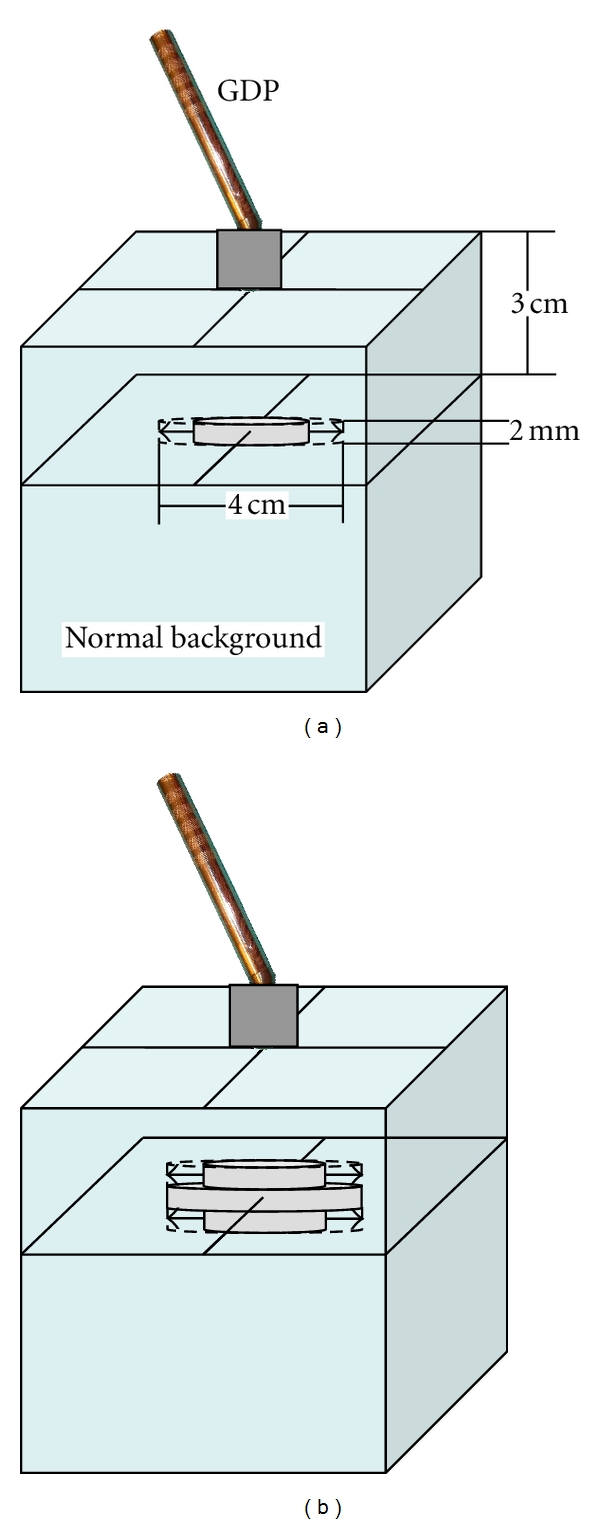
These figures illustrate the iterative growth of the tumour volume used to determine MDTV. (a) Stage 1: the grey disc represents the tumour volume, seen here growing horizontally to a maximum of 4 cm in diameter. (b) Stage 2: two new layers, each 2 mm thick, have been added vertically to the tumour volume, and horizontal growth will occur only in these new layers to a maximum of 4 cm in diameter. This process continues to a maximum length of 4 cm.

Using a computer simulation, the modelled tumour volume was iteratively increased in size and the detected counts were calculated. The point at which the expected counts exceeded the detection limit determined the MDTV for that combination of input parameters. The modelled tumour volume was increased using a region-growing technique that resulted in horizontal growth of the tumour prior to vertical growth (Figure 2). This represented a difficult scenario for a particular tumour volume, that is, a broad, flat tumour. In contrast, an algorithm in which a tumour grows horizontally and vertically simultaneously, like a sphere, would result in a smaller MDTV since the tumour would occupy a region closer to the detector where it is most sensitive. During BCS patients lay supine, which results in their entire breast flattening along with the cancerous lesion; therefore, spherical models are unlikely to represent the shape of tumours *in vivo*, accurately. 

### 2.3. Tumour Edge Delineation

Tumour edge delineation experiments were conducted *in silico* to assess the ability of the GDP to determine the edge of a tumour phantom in a uniform background. To facilitate comparison of all of the parameters being investigated, a simple tumour model was created. Generating a tumour model that captures all of the complexities of actual tumours is difficult; however, the model chosen in this study accounts for some of the main features that are important to the task of RIGS for lumpectomies [4, 5]. The tumour modelled had the shape of an oblate spheroid, with a maximum diameter of 3 cm and a length of 2 cm. This shape was chosen because it tapers towards the edges of the tumour, which in turn reduces the activity through the full thickness of the lesion towards its periphery, as would be expected in real tumours. Additionally, when patients are lying supine on the operating table, tumours tend to compress; therefore, a fully spheroidal shape would be less realistic. The tumour was modelled at a depth of 3 cm, which is representative of a depth where it is currently difficult to delineate the edges of the disease accurately by palpation. Measurements were taken assuming an anterior approach along the skin surface, as would be done *in vivo*.

Using this tumour model in conjunction with a uniform background activity, a 3D activity distribution was created for different combinations of normal tissue activity (derived from background activity concentrations) and tumour activity (derived from both normal tissue activity and T:B ratios). The response of the GDP to each distribution was calculated using the 3D point-response function discussed earlier. The measured edge was determined as the distance from the centre of the tumour model to the point where the calculated counts fell below the detection level. For a range of collimator lengths, background activity concentrations, and energy-window thresholds, the minimum T:B was determined that resulted in the location of the measured edge corresponding to the true edge of the tumour model. This represented the point where the GDP was able to accurately delineate the edge of the tumour model.

## 3. Results

### 3.1. Validation of the 3D Point-Response Function

The calculated 3D point-response function was experimentally validated by measuring the sensitivity profile of the C-Trak GDP at 1 cm depth, with a physical collimator that had a length of 0.5 cm and the energy-window threshold at the Compton back scatter peak for each of the radionuclides used in this study. The maximum deviation between the calculated sensitivity profile and the measured profile was 3.4%, indicating that the method to generate the 3D point-response function was accurate and was suitable for evaluating the physical parameters involved in RIGS.

### 3.2. Effect of Energy-Window Threshold on MDTV

**Figure 3:**
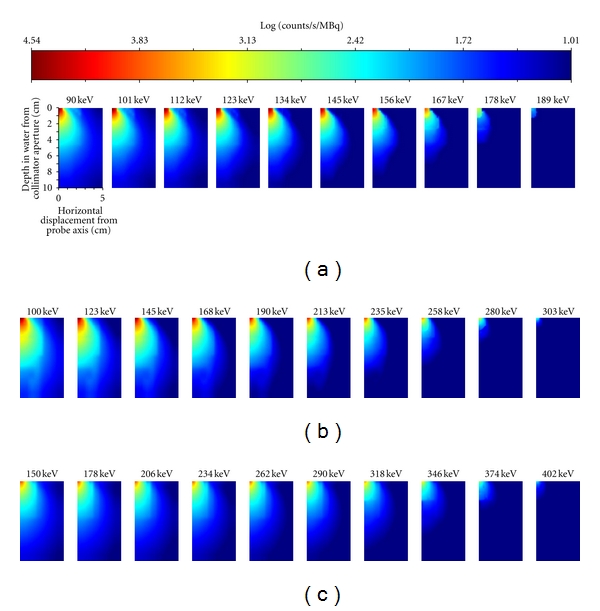
The effect of increasing the lower energy-window threshold on the sensitivity maps for (a) ^99m^Tc, (b) ^111^In, and (c) ^131^I. The lowest threshold was placed at the Compton backscatter peak for each radionuclide, and the upper threshold was placed at three standard deviations (derived by fitting a Gaussian distribution to the photopeak of the energy spectrum) above the photopeak. The top left corner of each image corresponds to a sensitivity measurement directly in front of the centre of the GDP, while the lower right corner of each image corresponds to a depth of 13 cm and a horizontal displacement of 7 cm.

Figure 3 illustrates the impact of increasing the energy-window threshold on the 2D sensitivity of the GDP for ^99m^Tc, ^111^In, and ^131^I sources, respectively. From these figures, it was observed that increasing the energy-window threshold reduced the sensitivity of the GDP. The decrease in sensitivity, however, was greater in areas that are further away from the central axis of the probe, where one would prefer the GDP not be sensitive (since off-axis sensitivity of this type would lead to poor spatial resolution).

**Figure 4:**
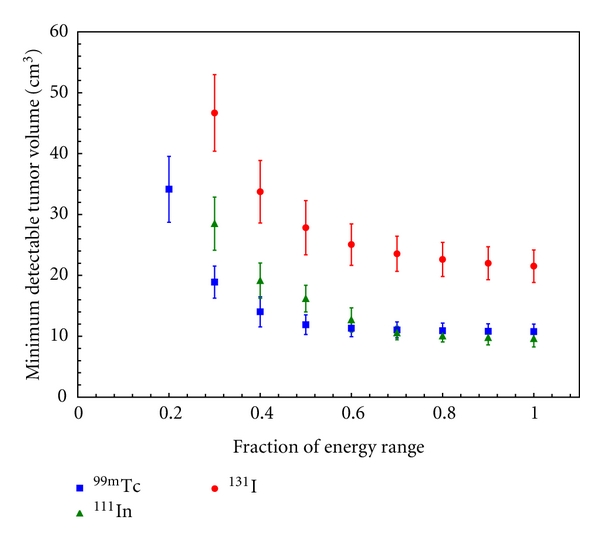
The effect of increasing the lower energy-window threshold on the MDTV for ^111^In, ^99m^Tc, and ^131^I. The T:B was fixed at 5. 0.0 cm collimation and a background activity concentration of 5 kBq/ml were used during these calculations to isolate the effect of increasing energy-window threshold on the MDTV. The abscissa of the plot corresponds to the fraction of the energy range that is above the energy-window threshold (0 corresponds to the threshold at  3 *σ* above the photo peak and 1 corresponds to the threshold at the Compton backscatter peak).

Figure 4 shows that for all three radionuclides increasing the energy-window threshold increases the MDTV. The error bars in Figure 4 are calculated by summing in quadrature the Poisson noise in the sensitivity map measurements and the variation in the volume of blood within breast tissue as published in [31]. However, the error bars did not incorporate variations in T:B as this was one of the parameters being evaluated (i.e., T:B). 

### 3.3. Effect of Collimator Length on MDTV

**Figure 5:**
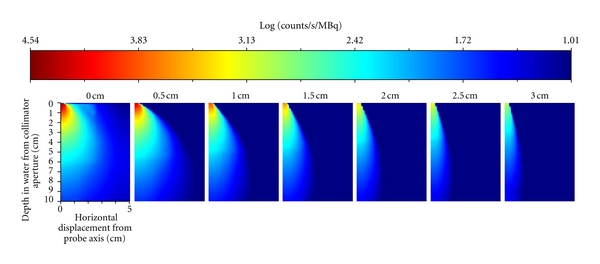
A graphical depiction of the impact of increasing the collimator length on the 2D sensitivity of the GDP for a ^99m^Tc  source from 0.0 cm to 3.0 cm. The upper left corner of each image corresponds to a sensitivity measurement directly in front of and at the midline of the GDP, while the lower right corner of each image corresponds to a depth of 10 cm and a horizontal displacement of 5 cm.

**Figure 6:**
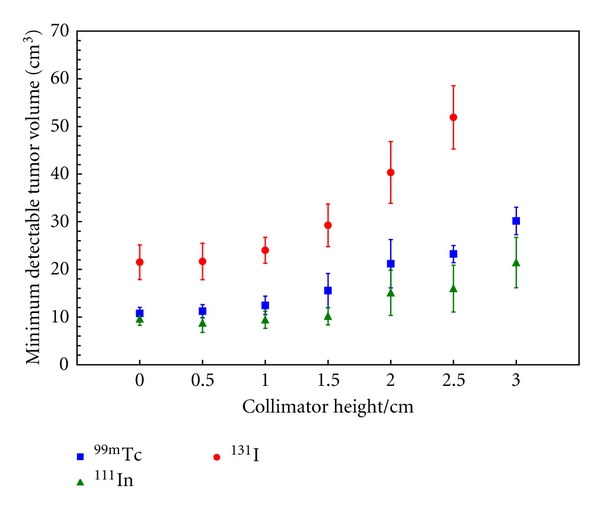
The effect of increasing the collimator length on the MDTV for ^111^In, ^99m^Tc, and ^131^I. The T:B was fixed at 5, the background activity concentration was fixed at 5 kBq/ml, the lowest energy-window threshold was applied for each radionuclide, while the collimator length was increased from 0 to 3 cm in increments of 0.5 cm. The error bars in this plot were calculated by summing in quadrature the Poisson noise in the sensitivity map measurements and the variation in the volume of blood within breast tissue [31].

Figure 5 illustrated the trend with which collimator length affected 2D sensitivity for ^99m^Tc, which is representative of all three radionuclides. These images display how increasing the collimator length decreases the field of view, thereby improving the spatial resolution of the system. As shown in Figure 5, the maximum sensitivity decreases rapidly as collimator length is increased.  Figure 6 plots the effect of increasing the collimator length on the MDTV for all three radionuclides. The increased collimator length places the detector further away from the radioactivity, resulting in a decrease in the sensitivity of the GDP in accordance with the inverse square law. This is apparent in the parabolic trends that are observed in the MDTV curves for each of the three radionuclide. The smallest MDTV for ^99m^Tc and ^131^I occurred when there was 0.0 cm of additional collimation extending beyond the sensitive face of the GDP. Similar trends were observed for ^99m^Tc when the tumour was placed at depths 1 cm, 2 cm, and 3 cm, which verified that the relationship between collimator length and MDTV was relatively unaffected by the tumour depth. For ^111^In, the smallest MDTV occurred when the collimator length was 0.5 cm.  Figure 6 shows that ^111^In consistently had the smallest MDTV; however, the differences in the smallest MDTV between ^111^In and ^99m^Tc were not statistically significant.

### 3.4. Effect of Background Activity Concentration on MDTV

**Figure 7:**
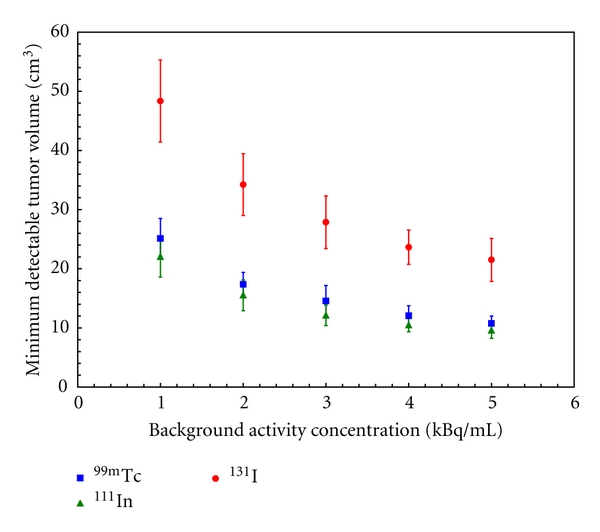
The effect of increasing the background activity concentration on the MDTV for ^111^In, ^99m^Tc, and ^131^I. T:B was fixed at 5, 0.0 cm collimation was used, and the entire energy range was used for acquisitions.

In Figure 7, it can be observed that the MDTV is inversely proportional to the square root of the background activity concentration. As the background activity concentration is increased, the slope of the MDTV curve decreases. The error bars in Figure 7 are calculated by summing in quadrature the Poisson noise in the sensitivity map measurements and the variation in the volume of blood within breast tissue as published in [31]. Under these experimental conditions, a background activity concentration below 3 kBq/mL significantly increases the MDTV compared to a background activity concentration of 5 kBq/mL. Both ^99m^Tc and ^111^In have statistically superior performances to ^131^I at all background activity concentrations (*P* < 0.05). There also appears to be no significant difference between the performance of ^99m^Tc and ^111^In. 

#### 3.4.1. Tumour Edge Delineation

For each combination of collimator length and energy-window threshold, the minimum T:B required for tumour edge delineation was determined at increasing background activity concentrations for each radionuclide. In this fashion, the effect of the combination of all of these parameters on tumour edge delineation was determined.

The parameters used to generate Table 2 resulted in the best performance of all combinations evaluated and were as follows: for ^111^In, a 100 keV energy-window threshold and a 0.5 mm collimator length; for ^99m^Tc, a 90 keV energy-window threshold and 0.0 cm collimation; for ^131^I, a 150 keV energy-window threshold and 0.0 cm collimation. The lowest T:Bs obtained were 6.2 ± 0.5 for ^99m^Tc, 5.2 ± 0.4 for ^111^In, and 12.0 ± 0.9 for ^131^I. All were obtained at the highest background activity concentration modelled of 5 kBq/mL. From Table 2, it is apparent that ^111^In and ^99m^Tc can be used to accurately delineate the tumour edge at all background activity concentrations at significantly lower T:Bs than is possible with ^131^I. In addition, there is a trend for ^111^In to have the lowest required T:B of the three radionuclides evaluated.

## 4. Discussion

In order to evaluate the feasibility of RIGS of breast cancer, the optimum system parameters were determined. This was found to be an ^111^In radiolabel for the highest background activity concentration evaluated (5 kBq/mL), a collimator length of 0.5 cm, and an energy window threshold of 100 keV, for detecting tumours in a uniform background at a depth of 3 cm. A tumour must be greater than 8.7 ± 2 cm^3^ (corresponding to a diameter of 2.5 ± 0.2 cm) to reduce re-excision rates from their current levels of 15–40% [2, 3] to 5% using RIGS at a clinically achievable T:B of ~5 [15].

*Radiolabel:* ^99m^Tc. As expected, the C-Trak GDP was more sensitive to ^111^In and ^99m^Tc than to ^131^I. The reason for this was the lower efficiency of the NaI(Tl) crystal used in the C-Trak GDP for the 364 keV photons emitted by ^131^I. Barber et al. [32] have shown that scintillation crystals such as NaI(Tl) are more sensitive than semiconductor crystals such as CZT and CdTe at the photon energies used in this study. The higher sensitivity for ^111^In and ^99m^Tc compared to ^131^I, therefore, should also be true for other commercially available GDPs. This study has shown that an ^111^In label results in the best performance; however, a counterintuitive finding from this study is that ^111^In and ^99m^Tc perform similarly when variations in blood volume within breast tissue are incorporated into the phantom model. For example, similar-sized tumours may be detected by using ^99m^Tc in a patient who has a high activity concentration in their blood as compared to using ^111^In in a patient with a low activity concentration in their blood. As ^111^In emits two photons, it would be expected to produce more counts per unit activity, which due to Poisson statistics would reduce the relative error in each measurement compared to ^99m^Tc. However, the two photons emitted by ^111^In differ in energy (171 keV and 245 keV), and the efficiency of the GDP for the 245 keV photon is low (75% of the efficiency for the 171 keV photon using a NaI(Tl) crystal 3 cm thick). Therefore, the overall benefit of having two photons per decay is diminished. Higher background activity concentrations of ^99m^Tc-labelled radiopharmaceuticals can be achieved for the same patient radiations dose as compared to ^111^In. Thus, it may be possible to detect smaller tumour volumes using ^99m^Tc. The half-life of ^99m^Tc (6 hours), however, is much shorter than that of ^111^In (2.83 days), limiting the use of ^99m^Tc  to targeting agents that have rapid clearance from the blood stream.

*Background Activity Concentration: 5 kBq/mL.* An increase in this parameter was assumed to cause proportional increases in normal-tissue background and in tumour activity concentrations. Under these conditions, it was demonstrated that the MDTV is inversely proportional to the square root of the background activity concentration for the assumed tumour model. As stated previously, background activity was assumed to relate directly to administered activity. In principle, the higher the lesion activity, which is associated with a higher background activity, the lower the statistical uncertainty in the measured count rates. This lower statistical uncertainty in turn helps to reduce the minimum detectable tumour volume. This suggests that administering more activity will result in the detection of smaller tumours, as was expected. In principle, the background activity concentration can be translated into administered activity for any radiopharmaceutical, taking into account its specific elimination pharmacokinetics. The radiation dose to the patient per unit of injected activity depends on the radionuclide and the pharmacokinetics of the agent involved. Assuming similar kinetics, the effective dose per unit administered activity dose for ^99m^Tc is considerably lower than those for ^111^In (which emits photons and Auger electrons) and ^131^I (which emits photons and beta particles). Since lower radiation doses to the patient would be expected for ^99m^Tc compared to ^111^In and ^131^I, it may be possible to administer more activity to patients by using a ^99m^Tc radiolabel; therefore, a higher background activity concentration can be achieved. Of course, ^111^In's long half-life has the advantage of allowing greater time for clearance of nonspecific uptake than does ^99m^Tc's  short half-life.

*Collimator Length: 5* *mm.* The lowest MDTV using ^111^In resulted when a 5 mm long collimator was used, whereas for ^99m^Tc and ^131^I 0.0 cm collimation was required. Further investigation of ^111^In showed that the increase in collimation decreases the number of photons from outside the field of view that penetrate the lead shielding of the GDP and are incorrectly detected. For ^111^In, 2 mm of lead is required to stop 95% of the 171 keV photons, while the 245 keV photons require 5 mm of lead. A 5 mm extension of the collimator is, therefore, required to provide adequate absorption of both photon energies and may explain why 5 mm collimation resulted in the lowest MDTV for ^111^In. For ^99m^Tc, 1.2 mm of lead is required to stop 95% of the 140 keV photons. The impact of a collimator extension of 1.2 mm could not be evaluated using the 3D point-response function since it had a spatial resolution of 2 mm. Due to the coarseness of the 3D point-response function, it was found that 0.0 cm collimation was better than a 2 mm collimator length. For ^131^I, 14.7 mm of lead is required to stop the 364 keV photons. Since the sensitivity of the GDP is already low for these photons, increasing the collimator length only degrades performance by further decreasing the sensitivity of the system. Changing the depth of the tumour from 1 to 3 cm did not alter the fact that increasing the collimator length, beyond the length required to properly shield the GDP, dramatically degraded the GDPs performance. Sensitivity of the GDP seems to be the dominant factor in determining the optimal performance of the system. 

*Energy-Window Threshold: Compton Backscatter Peak.* In RIGS the sensitivity of the system is more important than the spatial resolution; as such, the lowest MDTV results when the energy-window threshold was placed at the Compton backscatter peak, indicating that the improved spatial resolution from increasing the energy-window threshold did not compensate for the decrease in sensitivity.

*Tumour Edge Delineation:* This evaluation determined that a minimum T:B of 5.2 ± 0.4 is required in order to delineate a 3 cm diameter tumour from a uniform background using an ^111^In radiolabel, 5 mm collimator length, and a background activity concentration of 5 kBq/mL. The commonly held paradigm is that a long collimator with no energy-window threshold, thereby a system with high spatial resolution with the highest possible sensitivity, would best suit this application. Our findings show that this is not the case: the impact of collimation, while improving spatial resolution, increases the separation of the probe from the skin surface. This has two effects: (1) sensitivity drops dramatically due to inverse square law and (2) the change in sensitivity with increasing distance becomes less dramatic (Figure 5) resulting in the probe becoming relatively more sensitive to background radiation. Both of these properties make the extended collimator undesirable for RIGS. While it is true that with a 0.0 cm collimator much of the gross tumour will still be detected when the probe is positioned at the tumour edge, the inverse square dropoff in sensitivity from the centre of the probe acts like a virtual collimator, whilst still maintaining maximum possible sensitivity. 

Gamma-rays emitted by the radiolabels that are used for RIGS penetrate large thicknesses of tissue, and GDP readings are thus adversely affected by normal background counts distal to the probe. Therefore, to distinguish a tumour from background, the tumour must have a signal substantially larger than that from surrounding normal tissues; that is, the radiopharmaceutical must be more specific to the tumour. Creating radiopharmaceuticals that selectively target cancer cells, that do not bind to normal cells, and that have a rapid clearance from the patient is technically challenging.

More recently beta probes have been introduced as an alternative to GDPs. The technique of beta particle detection uses the short range of beta particles (on the order of millimeters in tissue) to its advantage, by reducing the detected signal from distant organs and tissues that would otherwise confound a conventional GDP. Additionally, the short range of beta particles precludes the need for collimation; thus, sensitivity is retained. The beta particles emitted by ^18^F, a commonly used radiolabel, have a range of 1-2 mm in tissue. This theoretically allows for localization of residual tumour to within 1-2 mm, making beta imaging probes potentially well suited to evaluating the postsurgical cavity to ensure clearance intraoperatively. This same property, however, renders beta imaging probes unsuitable for intraoperative tumour delineation, since the tumour would be excessively deep [33–38].

## 5. Conclusion

There are at least two key limitations in the manner in which potential radiopharmaceuticals for RIGS are currently evaluated: (1) the most appropriate set of system parameters has yet to be established; (2) no minimum T:B has been suggested as being required to determine whether a radiopharmaceutical is likely to be a good candidate for use in RIGS. The results reported in this study have shown that the most appropriate combination of parameters included a ^99m^Tc radiolabel with 0.0 cm collimation and minimal energy-window thresholding or ^111^In with a collimator length of 0.5 cm. Additionally, it was shown that a T:B of at least 5.2 ± 0.4 is required to accurately delineate the tumour edge of a 3 cm diameter lesion from a simple uniform background. Therefore, the role of RIGS for guidance of breast-conserving procedures is promising; however, novel radiopharmaceuticals need to be developed that have a higher specificity for cancerous tissue *in vivo* than what is currently available.

## Figures and Tables

**Table 1:** Published tumour-to-background activity concentration ratios achieved for radiopharmaceuticals used to target TAAs in breast cancer.

Tumour-associated antigen	Tumour-to-background activity (T:B)	Reference
^111^In-B72.3 (TAG-72)	4.3 ± 0.91^i^	[15]
^111^In-Trastuzumab (HER-2)	1.3 − 9.3^ii^	[19]
^111^In-DTPA-Trastuzumab F_ab_ (HER-2)	25.2 ± 1.62^iii^	[16]
^111^In-benzyl-DOTA-Z (HER-2)	17 ± 2.3^iii^	[17]
^131^I-CEA antibody	1.4 ± 0.3^ii^	[21]

^i^T:B ratio was determined using well chamber measurements of normal and cancerous tissue uptake in a series of mastectomy samples.^ii^T:B ratio was determined by gamma camera imaging of patients enrolled in a clinical trial, evaluating the tissue uptake in cancerous lesions and in background.^iii^T:B ratio was determined using well chamber measurements of normal and cancerous tissue uptake from xenografted sacrificed mice.

**Table 2:** Minimum T:B ± standard deviation to achieve tumour edge delineation. Background activity concentration is a surrogate for administered activity and can be related to relative error in GDP measurements. The combinations of parameters resulting in the optimum overall performance and used to generate the data in this table are as follows: for ^111^In, a 100 keV energy-window threshold and a 0.5 mm collimator length; for ^99m^Tc, a 90 keV energy-window threshold and 0.0 cm collimation; and for ^131^I, a 150 keV energy-window threshold and 0.0 cm collimation.

Radionuclide	Background activity concentration				
	1 kBq/mL	2 kBq/mL	3 kBq/mL	4 kBq/mL	5 kBq/mL
^99m^Tc	14.4±1.1	10.0±0.8	8.1±0.6	7.0±0.5	6.2±0.5
^111^In	11.9±0.9	8.3±0.7	6.7±0.6	6.0±0.6	5.2±0.4
^131^I	28.3±2.2	19.4±1.5	15.6±1.2	13.4±1.0	12.0±0.9
